# Applications of MCDM approach (ANP-TOPSIS) to evaluate supply chain solutions in the context of COVID-19

**DOI:** 10.1016/j.heliyon.2022.e09062

**Published:** 2022-03-09

**Authors:** Ghazi M. Magableh, Mahmoud Z. Mistarihi

**Affiliations:** Industrial Engineering Department–Yarmouk University, Irbid-Jordan Yarmouk University, P.O. Box 21163, Irbid, Jordan

**Keywords:** Supply chain, COVID-19 pandemic, MENA, ANP-TOPSIS, MCDM, SC solutions

## Abstract

COVID-19 has impacted various aspects of life and business. The economic panic and effects caused by the pandemic have led to disruptions in most supply chains (SCs) worldwide. Our research aims to analyse the impact of COVID-19 on SCs and enable organisations to prioritise solutions based on their relative importance. The research consists of two main phases. Phase I analyses the impact of the pandemic on SCs in terms of challenges, concerns, steps, and solutions with the goal of SC resilience. The second phase proposes a merged Analytic Network Process-Technique for Order Preference by Similarity to Ideal Solution framework (ANP-TOPSIS) to prioritise the solutions that considers the complicated interrelationships between the factors involved in decision-making. The proposed model increases the efficiency of the decision-making process and helps decision makers effectively select solutions based on their importance and impact on business. The results indicate that SCs should continuously utilise technology to withstand future competition and crises.

## 1 Introduction

COVID-19 affects the world economy and businesses as it spreads across the world and ongoing supply chains (SCs) have been hit hard, leading to a shortage in many critical products. The nature of COVID-19 has resulted in increased exposure of SCs to disruptions. Local and international SCs were affected but the pandemic has had a greater impact on international SCs. Changes in supply and demand along with the countries' actions to eliminate the spread of the virus have caused great disruptions to the global supply chain (GSC). As in any other region, the SCs in the Middle East and North Africa (MENA) region have been greatly affected.

The impact of the pandemic on SCs has become evident among decision-makers, especially with regard to the disruptions in supply and demand. Organisations realise the urgent need to identify appropriate solutions and indicate their impact on the future stability and performance of the SC. Since companies cannot implement all solutions at the same time, there is a need to prioritise these solutions according to their importance and effectiveness at reducing the impact of the pandemic on the SC and to ensure continuity in the flow of goods and retain future competitiveness.

In order to reach the appropriate results and after reviewing previous studies, a survey was conducted to study the impact of the COVID-19 pandemic on SCs in terms of its challenges, main concerns, and worries during the pandemic; the steps necessary to reach the new normal after the pandemic; and the solutions that could contribute to achieving SC stability and reducing the impact of disruptions. Additionally, a questionnaire was used to clarify each solution's significance and priority to determine the best solution(s) to achieve future SC resilience.

Evaluating and selecting SC solutions is a challenging problem because of differences in the type, size, location, and objectives of organisations and because of the inconsistent and varying experiences of decision makers. None of the traditional criteria makes the solutions less complicated. Furthermore, organisations cannot consider all solutions at the same time due to cost, time, and unavailability of expertise.

A merged multi-criteria decision-making (MCDM) approach that combines the analytic network process (ANP) and the technique for order preference by similarity to ideal solution (TOPSIS) method is used to rank the alternative solutions required to overcome the pandemic disruptions and reach SC stability and future resilience. The proposed framework merges the ANP and TOPSIS methods (ANP-TOPSIS) to rank the solutions from the first time and enable organisations to prioritise their solutions based on their relative importance.

The suggested model offers means of improving the efficiency of the decision-making process and assisting decision makers in selecting solutions more efficiently based on their importance and impact on business. Fuzzy MCDM methods like fuzzy ANP and fuzzy TOPSIS could also be used to cope with the ambiguous or imprecise nature of linguistic assessments. The ANP-TOPSIS framework is used during a crisis to address complex problems, evaluate interactions while allowing for complex interrelationships between decision-making elements, and select the optimal solution from a group of decision solutions and alternatives with numerous competing criteria (see sections 2, 5, 6 and 7). To illustrate how the merged framework is applied to the solution selection problem, real data was collected from the MENA region.

This study examines previous studies of the SC and COVID-19 in order to help SCs become more resilient in the future. It brings together previous studies and examines a variety of novel aspects and interconnections between distinct COVID-19 related SC concerns. Thus, the goal of this project is to provide answers to the following important research questions: What factors impact the SC's performance in terms of SCs' challenges, key concerns, and worries during the pandemic? What are the steps necessary to transcend the COVID-19 pandemic? What are the key solutions that contribute to achieving SC stability and resilience? How can an effective approach be incorporated into the decision-making process to boost its efficiency and assist decision makers in efficiently selecting solutions that are based on the importance of and impact on SCs? How can these solutions be prioritised for implementation given limited resources? The study aims to answer these questions, fill research gaps, and make the following contributions: expand current investigations to learn more about COVID-19's issues and concerns, determine the most effective options for and implications of SCs' long-term stability and performance, increase the efficiency of decision making by combining ANP and TOPSIS algorithms to rank alternative options, and verify the suggested method's rationality and applicability via a real-world case study of the MENA.

The remainder of this paper is organised in seven sections. Section 2 reviews previous work related to SCs and pandemics. Section 3 explains the research methodology. Section 4 discusses the main SC elements associated with COVID-19. Section 5 introduces the proposed framework in 5 main stages and 12 steps. Section 6 analyses and discusses the results. Section 7 presents the conclusion and future research prospects.

## 2 Literature review

COVID-19 has uncovered a series of SC operation and management challenges (Ivanov, 2020b). The interruptions the pandemic has caused are unusual events in the GSC and are characterised by high levels of uncertainty, longstanding disruptions and wide spread effects (Ivanov, 2020a). The social distancing mandated in response to COVID-19 has impacted global production and SCs (Shokrani et al., 2020). There is a major shortage in the production and supply of critical medical and health products (Iyengar et al., 2020). During crises, SCs have experienced supply disruptions, demand volatility, lack of preparedness, and deficiencies in existing response plans, but opportunities have also arisen for improving SC resilience (Remko, 2020).

SC-decision makers must build smarter and additionally resilient SCs for future flexibility. The pandemic has resulted in a clearer relationship between geopolitics and the SC decision-making process, a new age of the localisation of SCs, a less intermediate role for SCs, online delivery, and greater government intervention (Lopes de Sousa Jabbour et al., 2020). The main challenge is to design SC networks with sufficient resilience to withstand disruptions and maintain sustainability (Mari et al., 2014). The operation and management of SCs and operations during COVID-19 has consisted of several steps, including adaptation, digitisation, preparation, recovery plan implementation, mitigation of effects of a crisis, and SC sustainability (Queiroz et al., 2020).

The creation of sustainable SC organisations requires intelligent workflow, utilisation of smart SC tools such as AI and augmented reality, optimisation of supply networks, and a readiness for future disruptions and the new normal (Sharma et al., 2020). Sharing critical resources, managing risks, and ramping up manufacturing early in a pandemic can increase efficiency (Mehrotra et al., 2020). Employing 3D printers can help facilitate rapid development of custom made critical goods to cover SC shortages such as for urgently needed healthcare products (Cox and Koepsell, 2020).

Qualitative and quantitative evaluations of risk mitigation strategies are essential to prioritise solutions and practices (Rajesh, 2020). An ANP technique is a fast multi-attribute decision-making tool that enables the regulation of SC performance and integrates different alternatives into the decision model. It considers the interrelationships between hierarchies and is effective for understanding both qualitative and quantitative factors (Agarwal et al., 2006). The ANP technique is used to prioritise SC initiatives concerning the causal relationships between multiple decision variables (Masoumik et al., 2015). Like MCDM, the ANP method is used to offer a decision support model to measure the performance of the SC process (Moons et al., 2019).

TOPSIS is an MCDM method employed to find and prioritise the best solution from a set of alternative solutions using similarity. It is an evaluation tool used in decision-making to rank alternatives in different areas (Liu et al., 2017). It considers the interdependency among criteria (Vinodh, 2015) and is commonly utilised to rank order lean strategies (Prasad et al., 2018). TOPSIS considers the vagueness of decision-making and is employed to choose the best alternative solution using interrelated relationship criteria derived from a limited set of decision solutions. It is widely employed because it is easy to implement and logical.

A hybrid approach based on integrated ANP-TOPSIS has been proposed for assessing facility-layout selection (Zha et al., 2018), sorting out the best suppliers (Abdel-Basset et al., 2018), evaluating and selecting suppliers (Shahroudi and Rouydel, 2012), and selecting the best location for a service apartment when combined with the fuzzy Delphi method (Chang et al., 2015). ANP-TOPSIS combined with the multi-choice goal programming approach aids selection of a knowledge transfer strategy with an enterprise resource planning implementation (Cahyadi, 2019) and to select the strategy for launching new products onto the market (Liao et al., 2016). The combined ANP-TOPSIS approach is used to determine each criteria weight, develop a method to rank all alternatives, and select the best alternative while considering the subjective judgement of the experts and the complexity of decision-making in the selection process.

To the best of our knowledge, this is the first study to investigate solutions for SC disruption in the context of COVID-19 as well as to rank the proposed solutions for SCs in the MENA region. This research contributes to the enrichment of the literature by using realistic data from organisations and consulting experts in the region to identify and analyse challenges confronting the SCs, crucial concerns and worries during the pandemic, steps to reach the new normal post pandemic, and the solutions that contribute to achieving future SC resilience. The ANP-TOPSIS framework was used to rank alternative solutions and allow firms to prioritise their solutions depending on their relative importance. The model aims to increase the efficiency of the decision-making process, support decision makers in ranking solutions, and select the optimal solution(s). Moreover, the framework assists SC specialists in prioritising the solutions involving various criteria in other SCs and services.

## 3 Methodology and data collection

**Figure 1:**
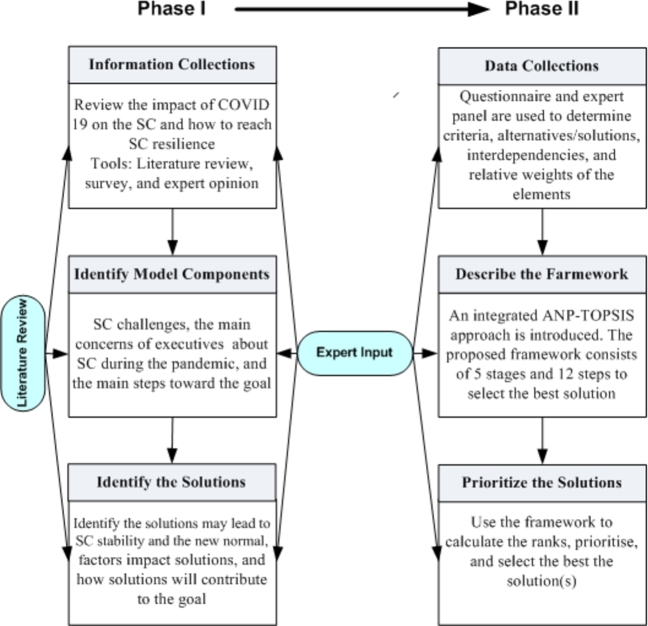
Research methodology.

The methodology in this study consists of two phases, as shown in Fig. 1. Phase one defines the goal, alternatives, solutions, and criteria. Phase two determines the main criterion, solutions, alternatives, and relative weights, and it solves the problem using the proposed merged ANP-TOPSIS method.

We define the main components of the model based on the literature review, public data, media and organisation reports, the opinions of specialists that have been published and propagated via the internet, and team discussions about the impact of COVID-19 on the SC. The components that include major challenges faced by the SC, steps toward future SC resilience, concerns or sources of worry to companies during the pandemic, solutions circulated between specialists and experts, and the main goal of SCs were identified and included in the survey. The survey was arranged in four sections, and a detailed description and information was provided to help respondents provide the appropriate data. Next, the survey was distributed to several organisations in the MENA, where ten SC specialists and executives responded to the survey.

In phase II, after studying and analysing the previous four parts separately (to define the interrelationship between them) and based on the results of the previous phase, the group of experts (two professors and three specialists in the field of SC management) determined the elements of each component to be considered in the calculations of the best solutions. A questionnaire was designed and distributed to the same respondents who were asked to specify the relative weights and subjective scores of each factor with respect to other elements. The elements included the criteria (challenges, concerns, and steps), sub-criteria as shown in Table 1, and alternative solutions for achieving the goal of future SC resilience. The collected data were then analysed using the ANP-TOPSIS framework to rank the suggested solutions. Finally, the results of the ranking of solutions in phase I were compared with the results of Phase II to validate and verify the effectiveness of the framework.

**Table 1:** Description of selection criteria.

Criteria (source[s])	Abr.	Definition
Demand volatility (Ivanov, 2020a; Remko, 2020)	DV	Unpredictable changes in demand
Supply disruptions (Remko, 2020; Ivanov, 2020a)	SD	Sudden crises that negatively impact the supply of items or services
Process interruptions (Liu et al., 2020; Rowan and Laffey, 2020)	PI	Disruptions in the processes and steps involved in SC activities to get a product or service to the customer
Government measures (Lopes de Sousa Jabbour et al., 2020; Magableh, 2021)	GM	The actions taken by the government to confront the pandemic
Personal safety concerns (Iyengar et al., 2020)	PS	General personal recognition and avoidance of possible harmful situations due to COVID-19
Safety (Abdel-Basset et al., 2018)	S	Worker safety or occupational health and safety due to the pandemic
Continuity (Lopes de Sousa Jabbour et al., 2020)	C	The continuity and stability of SC flows, such as material, financial, and informational flows
Quality (Iyengar et al., 2020)	Q	Quality of goods
Cost control (Remko, 2020; Magableh, 2021)	CC	Practices to maintain or lower costs during the pandemic
Time (Rowan and Laffey, 2020; Iyengar et al., 2020)	T	SC delays in time due to the pandemic
Stability (Liu et al., 2020)	ST	Reliable, predictable, and agile SC that is capable of delivering against customer requests
Cash flow (Magableh, 2021)	CF	Liquidity or the net amount of available cash in the organisation
Risk mitigation (Lopes de Sousa Jabbour et al., 2020; Ivanov, 2020a; Remko, 2020)	RM	Strategy/plan to prepare for and minimise the effects of threats during crises and disasters
Opportunity (Magableh, 2021; Liu et al., 2020)	O	Opportunities to increase attainment during a crisis
Customer satisfaction (Rowan and Laffey, 2020)	CS	Customers' satisfaction with the organisation's products and services
Assessment and planning	AP	Phases that support the development of the SC
(Guan et al., 2020)		
Capability building (Remko, 2020; Rowan and Laffey, 2020) (Ivanov and Dolgui, 2020)	CB	The ability of SC personnel to perform work efficiently and improve performance
Focus on supply network	SN	Focus on the processes for adding value for customers via the manufacturing and delivery of goods
Digital collaboration (Ivanov, 2020b)	DC	A tool that utilises digital technologies for cooperation with partners to facilitate collaboration both in-office and remotely
Build SC resilience (Ivanov and Dolgui, 2020; Ivanov, 2020b)	BR	SC's preparedness for unexpected risks events and to respond and recover quickly from the effects of a crisis

## 4 COVID-19 pandemic and the supply chain (SC)

This section discusses and analyses the interconnections between the COVID-19 pandemic and the SC based on the literature review and the results of the survey from phase I. The results were analysed to determine the criteria (challenges, concerns, and steps), alternatives (solutions), and main goal for achieving SC resilience and sustainability.

### 4.1 Part I: challenges

This section analyses the impact of the COVID-19 pandemic on SCs in terms of the challenges and key causes of disruption, the phenomena associated with these challenges, the cause of each phenomenon, and their possible impact on the SCs. There are five main challenges that affect supply, distribution, production, and SCs activities and operations: supply disruption, demand volatility, process disruption, government measures, and personal safety concerns. Based on the responses that have been received, the main phenomena associated with these challenges, their possible causes, and their impact on SCs have been identified in Table 2.

**Table 2:** Phenomenon-cause-impact interrelationships.

Phenomenon	Possible Cause	Impact/effect
Production disruptions	Social isolation and distancing, government protection measures, discontinuation of supply, border closures, and transportation problems	Business disruptions; contraction of international trade flows; delivery challenges; unavailability of materials, components, finished products, and items used in factories; and increased costs
Panic buying	Demand spikes, market instability, social isolation and distancing, government protection measures, customers' stockpiling, and fear of personal safety	Stock out, price increases, and inconsistent demand quantity
Prices hikes	Production disruptions, banning exports, demand spikes, freight price spikes, abandonment of travel, extra orders, pressures on organisation to cut workers and production, volume decline, transit delay, lack capacity, and delivery delay	Price fluctuations; negative effects on the relations between suppliers, retailers, and customers; increase in demand for key products; decrease on demand for luxury products; and cancelled orders
Fraud	Demand spikes, low production, price increases, cyberattacks, increased opportunities for fraudsters to fill gaps	Low-quality goods and dissatisfied customers
Freight disruptions	Suspended transportation, ambiguity of routes, increased rates, uncertainty of import guidelines, delays in shipments, moving cargo, loading, shipping, unloading, and extra delay at the borders and ports	Fluctuations in transport costs, long lead time, delays in distribution, and delay in communication with sources and shippers
Supply, distribution, and delivery difficulties	Lack of raw materials, items, and components; production disruptions; shortage of inventory and storage; increased online orders; and government protection measures	Reduction in customer satisfaction, low inventory, price increases, inability to fulfil commitments, low responsiveness, limited ability to deliver, re-staffing of DCs and WHs, and changes in allocations of inventory across the network and distribution channels to increase responsiveness
Online shopping	Delivery constraints, direct distribution difficulties, social isolation and distancing, and personal safety concerns	Increased business for many online shops, decreased direct purchases, and increased pressure on online delivery
Stockpiling	Retaining goods and selling them later at inflated prices, panic buying, personal safety issues, and government protection measures	Stock out at stores (empty shelves), low inventory and storage, and price increases
Social isolation and distancing	Government protection measures, personal safety, and measures to limit the spread of COVID-19	Lockdown, suspended operations, factory closures, border restrictions, travel bans, port closures, suspended transportation, labour shortage, migration due to lockdowns and safety concerns, banning exports and sometimes imports, and abandonment of travel
Localisation trends	Trade barriers, supply challenges, border restrictions, and government protection measures	Increased focus on regional and local SCs
Reduction in quality measures	Low production; talent constraint; lack of inward quality control; reduction in production capacity, manufacturing capability, and the direct impact on sources of production; and demand spikes	Increases in fraudulent activities, decreases in customer satisfaction, and weak trust from consumers
Cash flow and liquidity constraints	New payment terms; flows disruptions; supply, production, and delivery constraints; social isolation and distancing; late payments; cancelled credit lines; inconsistent demand quantity; and increased costs	Panic to cut costs and generate cash, increased loans, cancelled orders, and rescheduling of payments and contracts
Unpredicted practices	Inefficient response to technology trends, panic to secure national supply, new legal issues and instructions, and nervousness regarding government decisions, rules, and regulations	Decreases in performance and disruptions of operations and activities
Reduction in return	Decrease in production; supply and delivery problems; disruption of SC activities, operations, processes, and their management; and government protection measures	Decreases in cash and liquidity, increases in employees layoffs, and decreases in business
Lack of crisis plans	Reduction in data access, difficulties in getting information and data from partners, lack of disruptions plans, small inventory levels, single supplier or minor diversification, underestimating the possibility of severe disruptions, focus on the short term and costs minimisations, lack of end-to-end visibility, lack of integration and coordination with SC partners, variations in technology utilisation, lack of SC risk management, lack of disruption mitigation plans, and lack of business strategies during crisis	Disruptions in planning, decision-making, capability, and visibility; lack of risk information and a contingency plan, increased awareness of reliance on multiple suppliers and sources, increased stock size to buffer against SC instability, and greater concerns about region or country, including local/domestic suppliers, factories, and industry

### 4.2 Part II: concerns

The second part aims to explain the main SCs concerns and worries of companies during the pandemic period and arrange them according to their importance. Based on the respondents' results, interest has been divided into three levels. The first level represents the direct immediate or the short term, the second level represents the medium term, and the third level represents the long-term concerns if the pandemic continues.

**Figure 2:**
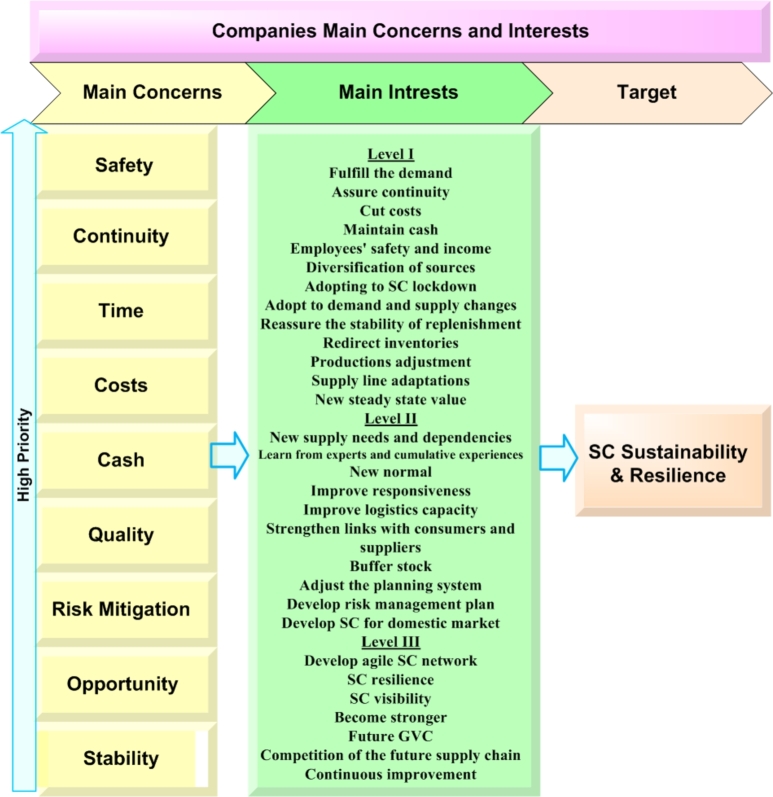
Rank order of the main concerns and their interests.

Depending on the answers from the survey, the concerns were arranged in descending order according to their priorities. As shown in Fig. 2, employee, personal, and public safety received the highest priority. Safety was prioritised because of the need to ensure that workers and employees were not infected, the disease did not spread further, and the productivity of factories, where the infected required treatment and quarantine for a relatively long period of time, did not decrease.

Continuity of flow across the SCs is an important priority for ensuring receipt of goods, especially critical items related to food and medical products. One of the most critical factors for SC competitiveness is the proper timing of delivery, as it is greatly affected by the lockdown and safety measures. SCs suffer from longer lead times due to delays in supply, distribution, shipments, and transportation, as well as additional delays at the borders and ports because of protection measures.

High prices and availability of cash are among the most important consequences for businesses. Negative repercussions on the SC include forcing companies to take precautionary measures to control prices, providing cash so that the relationship with customers does not worsen and ensuring the smooth flow of materials and goods. Cost control and management are crucial to SCs to confront demand variations and financial volatilities.

As a result of failure to take appropriate quality assurance and quality control measures in examining products, goods and the issues resulting therefrom, customer satisfaction and quality of the products offered have been significantly affected.

Companies seek to reduce risks in the SC to reach SC stability. Companies must analyse the potential risks locally, regionally, and internationally to determine the direct actions, plans, and strategies required. Risk factors are connected with geographical location, social issues, government regularities, and uncertainties in global business. Risk management should include safety measures. Moreover, if remote work continues, it will alter the risk focus.

The pandemic had provided unprecedented opportunities to enhance SC performance and stability. The extent of the opportunity depends on an organisation's capacity to handle risk better than it has before the pandemic. Remote work should receive attention because it may create new opportunities. Other opportunities include restoring plans and strategies, increasing the use of appropriate technologies, increasing competition and market share through creativity, diversifying dependence, using innovative methods of distribution, use of internet or online shopping, and increased digitisation. It is time for SCs to seize the new opportunities and improve their responsiveness, competitiveness, market share, and performance.

### 4.3 Part III: steps

**Figure 3:**
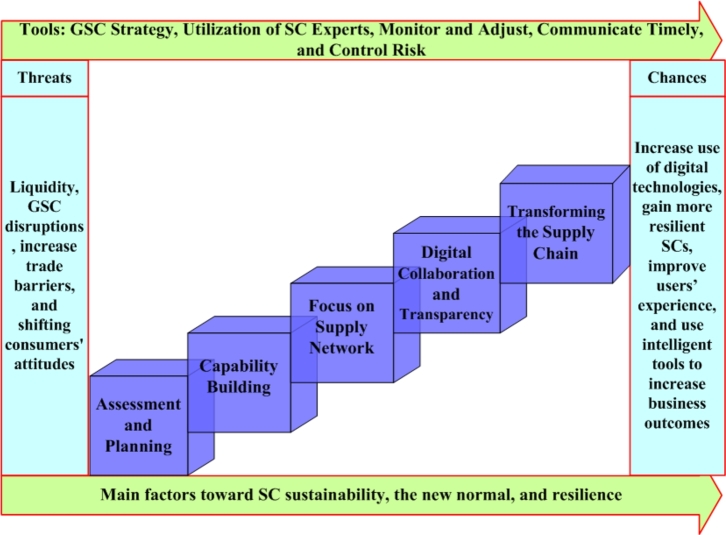
Main steps towards the new normal and SC resilience.

Based on the answers obtained from the survey, five main steps have been identified to achieve the SCs targets, new normal, and future resilience. These steps include, as shown in Fig. 3, assessment and planning, capability building, focus on the SC network, digital collaboration and transparency, and transforming the SC. The main threats facing SCs are liquidity, GSC disruptions, increase in trade barriers, and shifts in consumers' attitudes. On the other hand, the pandemic provides many opportunities for organisations to increase the application of digital technologies, improve SC resilience by using new technologies, and applying intelligent tools to increase business outcomes. Organisations should employ several tools to achieve SC targets, such as complying with global SC strategy; utilising SC experts to find innovative solutions; assessing, monitoring, and adjusting to disruptions; controlling risk; and communicating with suppliers, partners, and customers on a timely basis. Considerations of steps, threats, opportunities, and tools are the main factors of SC sustainability, the new normal, and resilience.

### 4.4 Part IV: solutions

In this section, solutions are identified that help SCs overcome the current crisis and lead to achieving the main goals of the GSC. The percentages (how solutions contribute to the goal) of each of these proposed solutions was determined by the respondents. Solutions, factors that contribute to achieving the solutions that lead to SC stability, resilience, and new normal are shown in Table 3.

**Table 3:** SC solutions and the main elements contributing to solutions.

Solutions	Factors contributing to the solution
Technological solutions	3D printers, IoT, predictive technology, cloud computing, big data and analytics, autonomous systems, industry 4.0 technologies, augmented realty, blockchain technology
Diversifications	New reliable suppliers, supply from closer sources, direct suppliers, optimising and managing supply networks
Digitisation	Emerging technologies, integration, transparency
Automation	Technologies, smart sensors, robots, unmanned vehicle systems, IT systems, communication advances
Visibility	Technology, blockchain, value chain, information sharing, automation, industry 4.0, digitisation, cumulative data from third parties, IT capabilities, digital collaboration
Integration	Partners' commitment, sharing transparent information, distribution of benefit, collaboration and cooperation, employing technology
Network agility	Advanced sensing, accessibility, flexibility, coordination, velocity, IT systems, new technology
Empowered teams	Empowered to be creative, professionally competent, using best practices, autonomous, possessing appropriate knowledge and skills, concerned about health and wellness, concerned about finances (social security), possessing leadership skills, engaged in self-discovery, entrepreneurial, possessing clarity regrading goals
Government intervention	Government support, regularity, reduction of trade restrictions (export, import, tariff taxes), cash-based assistance, strengthened healthcare at borders and ports to guarantee continuity, contributing to sustaining innovation efforts and existing productive capacity, financial system support, ensuring market conditions, security, transparency, connection between local factories and suppliers, reducing restrictions, investing in key SCs

**Figure 4:**
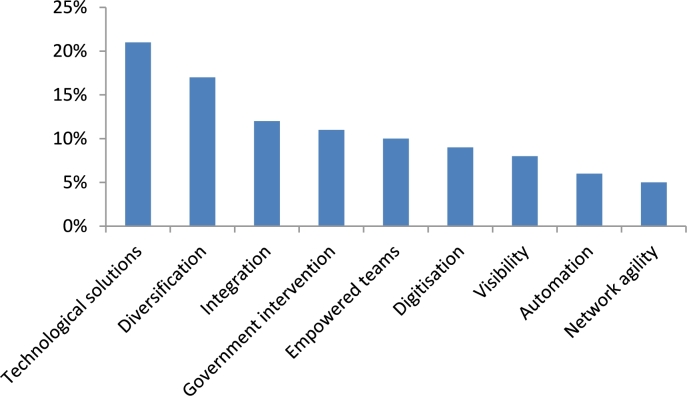
Rank order of solution elements that contribute to the SC goals of resilience and stability.

The target of organisation during the pandemic is to achieve a new normal and a resilient SC. The main objectives of a SC are to improve value for customers, efficiency, and responsiveness via the whole SC. It is clear from Table 3 that technology is a common factor contributing to all solutions. Based on the survey analysis, Fig. 4 shows the rank order of the solution elements.

## 5 Framework

In this section, the proposed ANP-TOPSIS framework is presented in detail. The framework consists of 5 stages and 12 steps to rank the suggested solutions. To clarify the applications and effectiveness of the techniques employed, the following is an overview of the two methods used in the *merged ANP-TOPSIS* approach.

### 5.1 ANP method

Complex decision-making for real-world problems requires the consideration of interdependency between problem components. The ANP method was developed by Saaty (Saaty, 1996, 1999, 2006) to consider the interactions and allow for complex interrelationships between decision-making elements. It structures the problem as a network where there are connections among criteria, sub-criteria, goals, and alternatives. The interaction between criteria and sub-criteria shapes the network and can solve interdependency by finding the relative significance of different criteria. The ANP method is split into the following process:

*Step 1: Assess criteria, construct network model, and structure problem*

**Figure 5:**
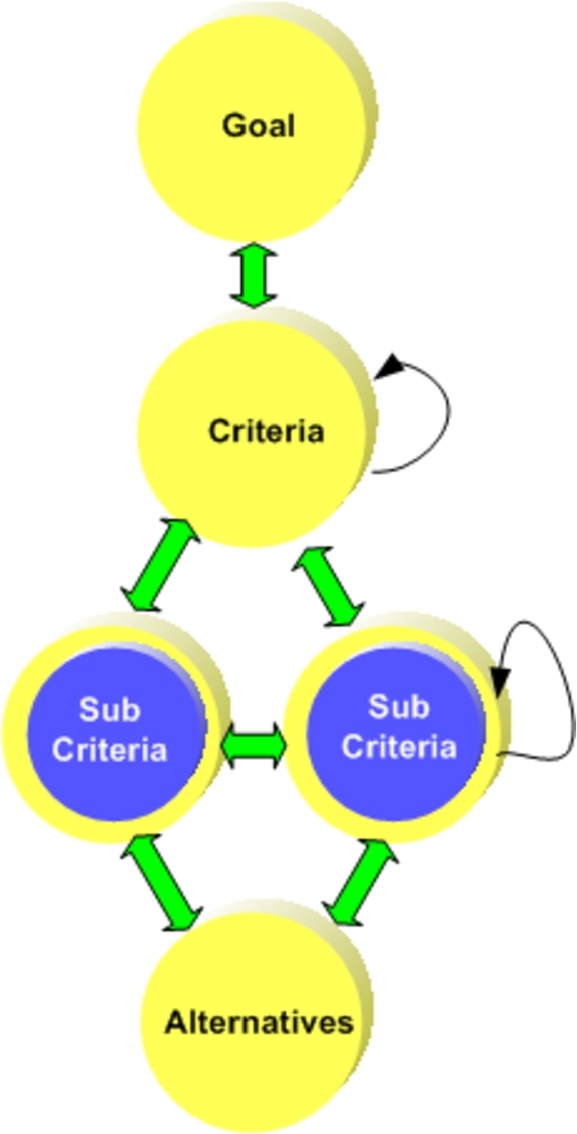
Network model.

The breakdown of the complex system into a logical system as a network requires the gathering of evaluation criteria, stating the problem, constructing the network structure based on experts' opinions and literature searches and analysing the interdependencies between criteria. In this step (as shown in Fig. 5), the MCDM problem is structured as a network and involves defining the elements, criteria, sub-criteria, and alternatives. It involves the assembly of an expert panel to capture the interdependency among criteria. Furthermore, feedback among network components is considered in this step.

*Step 2: Construct pairwise comparison matrix and the relative weights*

The decision-making panel performs a series of pairwise comparisons to create the relative importance of criteria. In the comparison, a 1 to 9 scale is used to compare the criteria based on the interdependency of the clusters and criteria. The number of comparisons *N* can be calculated as =n(n−1)/2. The eigenvector of the pairwise comparison matrix is used in the super matrix. The general form of the pairwise comparison matrix is as follows:

(1) P=C1C2…CnC1[1p12…p1n]C21/p121…p2n⋮⋮⋮⋱⋮Cn1/p1n1/p2n…1

Where $p_{ij}$ represents the relative importance of the element *i* against element *j*, i=1,2,…,m; j=1,2,…,n.

*Step 3: Consistency check*

Check the consistency property of the comparison matrix (*CI* and *CR*), which influences the effectiveness evaluation. The consistency ratio (*CR*) is acceptable when CR≤0.1, if not, there is a need to revise the comparison matrix. *CI*: Consistency index, *RI*: random index, $\lambda_{\max}$: maximum eigenvalue and *n*: number of criteria are related as follows:

(2) $$\mathrm{CR}=\frac{\mathrm{CI}}{\mathrm{RI}}$$

(3) $$\mathrm{CI}=\frac{\lambda_{\max}-n}{n-1}$$

*Step 4: Form and solve the super matrix*

Construct an unweighted super matrix. The unweighted super matrix is a matrix where each sub-matrix compromises a set of relations among clusters. The unweighted super matrix includes only the indirect influence, but not the intermediate elements, with the effect between a pair of elements. The resulting matrices are used to determine the impact of the relations among interdependent criteria. Next, a normalisation process is implemented to obtain the weighted super matrix. We then calculate the normalised eigenvectors of the pairwise evaluation matrices. Zero values of the eigenvector represent the independence of each other. The rest of the values represent the relative influence for each criterion. The general form of the super matrix is as follows:

(4) W=C1e11e12…e1m1C2e21e22…e2m2Cnen1en2…enmnC1e11e12⋮e1m1[W11W12W1n]C2e21e22⋮e2m2W12W22W2nCnen1en2⋮enmnWn2Wn2Wnn

where $c_{m}$: criteria *m*; $e_{\mathrm{nm}}$: element (sub-criteria) *n* in the criteria *m*; $w_{ij}$: principal eigenvector of influence of $j^{\mathrm{th}}$ element with $i^{\mathrm{th}}$ element. $w_{ij}=0$ if $j^{\mathrm{th}}$ element has no influence on the $i^{\mathrm{th}}$ element.

The normalisation of the direct relation matrix weights is given to make the sum of each column equal unity (=1). The value of each element in the matrix *Y* falls between 0 and 1 (0≤Y≤1).

(5) Y=kZ

(6) $$k=\frac{1}{\max_{1\leq i\leq n}\left(\sum_{j=1}^{n}z_{ij}\right)}\;\left(i,j=1,2,\ldots ,n\right)$$

*Step 5: Compute the limited matrix*

To obtain the limiting matrix, we raise the weighted matrix to a higher power until the row and column values of the super matrix are equal. The limit is achieved by increasing the weighted super matrix to a large power *k* until the weighted super matrix has converged and becomes stable to attain the global priority vector (ANP-weights). The general weight is calculated using the previous steps to obtain a stable supermatrix.

(7) $$\lim_{k\to \infty }w_{w}^{k}$$

*Step 6: Choose best alternative based on weight values*

According to the weights of solutions and alternatives with respect to the criteria in the limit matrix, the overall weight of each solution is calculated. Then, alternatives are ranked based on their total weights.

### 5.2 TOPSIS method

TOPSIS method is applied to calculate the total score of each alternative. It is used to select the best solution from a set of decision solutions and alternatives with multiple conflicting criteria. The MCDM-TOPSIS method consists of the following steps:

*Step 1*: Build the evaluation matrix of the alternatives and criteria, m×n where the elements in the matrix are denoted by $x_{ij}$. The decision matrix (*D*) of criteria and alternative values, where $x_{ij}$ represents the performance of criteria $C_{i}$ for alternative $A_{i}$ where i=1,2,…,m, and j=1,2,…,n is given as:

(8) D=C1C2…CnA1[x11x12…x1n]A2x21x22…x2n⋮⋮⋮…⋮Amxm1xm2…xmn

*Step 2*: Calculate the normalised value $x_{ij}$ where a normalised matrix is as:

(9) $${\bar{X}}_{ij}=\frac{X_{ij}}{\sqrt{\sum_{j=1}^{n}X_{ij}^{2}}}\;i=1,2,\ldots ,m;\;j=1,2,\ldots ,n$$

*Step 3*: Form the weighted decision matrix and calculate the weighted-normalised value $v_{ij}$. $w_{ij}$ Represent the weight for each criterion and is taken from the ANP limit super matrix. The weighted normalised matrix is calculated as:

(10) $$V_{ij}={\bar{X}}_{ij}XW_{j}$$

where $W_{j}$ denotes the weight of criteria *j* as in ANP and $\sum_{j=1}^{n}w_{j}=1$.

*Step 4*: Calculate the ideal positive $V^{+}$ and ideal worst values $V^{-}$ (Abdel-Basset, Mohamed, and Smarandache 2018)

$$V^{+}=\text{distance between the alternative and the positive reference point is considered as the minimum value of elements.}$$

$$V^{-}=\text{The distance between the alternative and the negative reference points is considered as the maximum value of the elements.}$$

(11) $$V^{+}=\left\{(\max V_{ij}\left|j\in J),(\min V_{ij}\right|j\in J)\right\}=\left\{V_{1}^{+},V_{2}^{+},\ldots ,V_{n}^{+}\right\}$$

(12) $$V^{+}=\left\{(\min V_{ij}\left|j\in J),(\max V_{ij}\right|j\in J)\right\}=\left\{V_{1}^{-},V_{2}^{-},\ldots ,V_{n}^{-}\right\}$$

(13) $$A^{+}=\left\{V_{1}^{+},V_{2}^{+},\ldots ,V_{j}^{+},\ldots ,V_{n}^{+}\right\}=\left\{(\max_{i}v_{ij}\left|j\in J)\right|i=1,2,\ldots ,m)\right\}$$

(14) $$A^{+}=\left\{V_{1}^{-},V_{2}^{-},\ldots ,V_{j}^{-},\ldots ,V_{n}^{-}\right\}=\left\{(\min_{i}v_{ij}\left|j\in J)\right|i=1,2,\ldots ,m)\right\}$$

Where $V^{+}$ represents the beneficial effect and $V^{-}$ represent the unfavourable effect.

*Step 5*: Calculate the distances to the positive $S_{i}^{+}$ and negative $S_{i}^{-}$ ideal solutions. The Euclidean distance is calculated as follows, where the range of every weighted element is between (0,1):

(15) $$S_{i}^{-}=\sqrt{\left[\sum_{j=1}^{n}{\left(V_{ij}-V_{j}^{-}\right)}^{2}\right]}\;i=1,2,\ldots ,m$$

(16) $$S_{i}^{+}=\sqrt{\left[\sum_{j=1}^{n}{\left(V_{ij}-V_{j}^{+}\right)}^{2}\right]}\;i=1,2,\ldots ,m$$

*Step 6*: Calculate the rank index. The relative closeness of the ideal solutions is calculated as follows: $0\leq P_{i}\leq 1$ i=1,2,…,m:

(17) $$P_{i}=\frac{S_{i}^{-}}{S_{i}^{+}+S_{i}^{-}}\;i=1,2,\ldots ,m$$

*Step 7*: Rank the alternatives in a descending manner, where the highest alternative score has rank 1. Then, consider the final decision based on the rank of alternatives. A higher index value represents the closer ideal solutions of alternatives.

### 5.3 Merged ANP-TOPSIS approach

A combined MCDM approach using the ANP-TOPSIS approach is used to rank the suggested solutions. The ANP-TOPSIS framework is utilised to solve the complex problem and find and rank the solutions to reach a stable SC during a crisis. The proposed framework of the combined ANP – TOPSIS consists of five main stages, where each stage contains several steps.

#### 5.3.1 Stage I: understand the multifaceted problem

To understand its interdependencies, the problem is defined, broken down, analysed, and discussed thoroughly with experts. This stage consists of the following three main steps:

*Step 1*: Select *m* experts to participate in making decisions. The decision group or board = $\left[e_{1},\;e_{2},\ldots \;e_{m}\right]$. The experts were asked to set the level of direct influence between the factors, denoted as $z_{ij}$ based on pair comparison. Since we have *m* expert opinions, the average was taken using the average value

(18) $$Z=\frac{1}{m}\sum_{d=1}^{m}z_{ij}^{d}$$

*Step 2*: Identify the decision criteria using the questionnaire, literature review, group discussions, brain storming, and/or media reports and examine these criteria with experts.

*Step 3*: Confirm both the alternatives and criteria of the model with the team of experts. Then, we determine the goal, solutions, and criteria for further examinations.

#### 5.3.2 Stage II: develop the model network structure

*Step 4*: Develop the ANP-model network using the solutions and criteria. Determine the interdependencies of the various criteria and start the construction of the network. The relationships among the criteria network should be examined.

#### 5.3.3 Stage III: ANP method

*Step 5*: Determine the relative importance of the criteria. All respondents and experts evaluated the criteria pairwise in a (1-9) scale, supposing that there is interdependency between them. We then determined the local weight of the criteria using pairwise comparisons. The priority matrix in terms of $w_{i}$ and $w_{j}$, where $w_{i}$ is the priority of element *i* is as below. Pw=nw and $w=\left(w_{1},\;w_{2},\ldots ,w_{n}\right)$.

(19) P=C1C2…CnC1[w1/w1w1/w2…w1/wn]C2w2/w1w2/w2…w2/wn⋮⋮⋮⋮Cnwn/w1wn/w2…wn/wn

*Step 6*: Calculate the pairwise comparison matrix of criteria regarding the goal. Further, check the consistency of the matrix.

*Step 7*: Calculate the internal interdependency of the main criteria according to each sub-criterion. Then, calculate the weight of each criterion based on the inner interdependencies ($w_{\mathrm{inner}}$). Calculate *CR* for each matrix. Then, calculate the normalised relative impact of the decision criteria ($w_{c}$: inner criteria weight)

(20) $$w_{c}=\left[\begin{array}{c} \mathrm{CH}\\ \mathrm{CO}\\ \mathrm{KS} \end{array}\right]=\left[\begin{array}{c} C_{11}\\ C_{21}\\ C_{31} \end{array}\begin{array}{c} C_{12}\\ C_{22}\\ C_{32} \end{array}\begin{array}{c} C_{13}\\ C_{23}\\ C_{33} \end{array}\right]x\left[\begin{array}{c} C_{1}\\ C_{2}\\ C_{3} \end{array}\right]=\left[\begin{array}{c} w_{\mathrm{ch}}\\ w_{\mathrm{co}}\\ w_{\mathrm{ks}} \end{array}\right]$$

*Step 8*: Calculate the comparison matrix for the local weights ($w_{\mathrm{local}}$) of criteria according to their clusters/sub-criteria.

*Step 9*: Calculate the sub-criteria global weights. The global weight for each sub-criterion is calculated using the formula:

(21) $$w_{\mathrm{global}}=w_{\mathrm{local}}\times w_{\mathrm{inner}}$$

#### 5.3.4 Stage IV: TOPSIS method

*Step 10*: Calculate the weighted normalised decision matrix. To form the standardised and evaluated weighted matrix, the decision-making group establishes the evaluation matrix by comparing the solutions with respect to each criterion. Next, the criteria weights taken from ANP are multiplied by the standardised evaluated matrix to obtain the standardised evaluation matrix.

*Step 11*: Calculate the closeness coefficients. Calculate positive and negative solutions and separation measures, as discussed in the TOPSIS method.

#### 5.3.5 Stage V: ranking alternatives

*Step 12*: Calculate the ranks of alternatives. Then, rank the solutions based on their score and select the best solution(s) with the highest score(s).

## 6 Results and discussion

The following steps summarise the calculations based on the ANP-TOPSIS framework.

*Step 1*: The team of experts consists of two professors and three specialists in the field of logistics systems and SC management. Ten executives responded to our questionnaire and filled the relative weights of the elements. The average was taken; for example, $p_{13}=5$ represents the relative importance of the element $C_{1}$ against element $C_{3}$ with respect to the goal, and is calculated as = (7+5+4+3+5+4+6+5+7+2)/10≈5.

*Step 2*: Based on the survey results, the decision criteria were selected, examined and determined with the experts. The selected criteria, their abbreviations (Abr), and their definitions are shown in Table 1.

**Figure 6:**
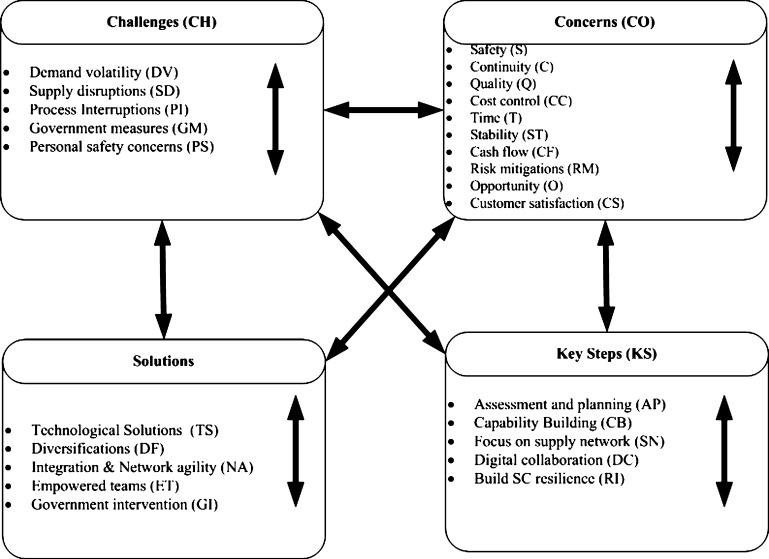
Model elements and their interdependent relationships.

*Step 3*: The alternatives and criteria of the model and their interrelationships and interdependency were confirmed by the team of experts as shown in Fig. 6.

**Figure 7:**
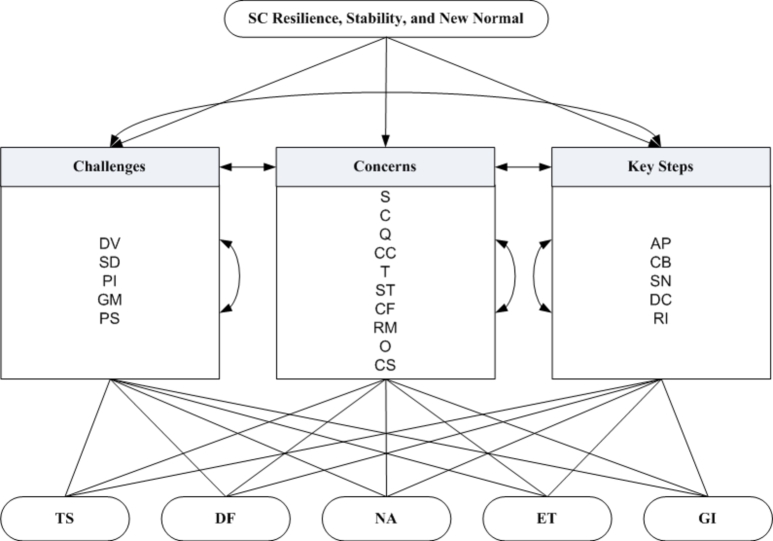
Network structure for the selection of best SC solutions.

*Step 4*: Fig. 7 represents the examined ANP-model network with the interdependencies of the different clusters and elements.

*Step 5*: Based on the responses, the relative importance of the criteria, sub-criteria, and solutions were specified. The average values of the comparison matrices are presented in Table 4, Table 5, Table 6. In Table 4, the values above the matrix diagonal (0.34, 5, and 7) represent the average values taken from the respondents as described in step 1. The other values (3, 0.2, 0.14) represent the inverse values of the elements above the matrix diagonal, for example, $p_{31}=1/p_{13}=1/5=0.2$.

**Table 4:** Criteria–goal matrix.

Goal	CH	CO	KS
CH	1	0.34	5
CO	3	1	7
KS	0.2	0.14	1

**Table 5:** Standardised criteria–goal matrix.

Goal	CH	CO	KS	Weight
CH	0.24	0.23	0.38	0.28
CO	0.71	0.68	0.54	0.64
KS	0.05	0.09	0.08	0.07
$\lambda_{\max}=3.06$; CI = 0.029; CR = 0.05

**Table 6:** Internal interdependency (Main Criteria-CH) matrix.

**CH**	CO	KS	Weight
CO	1	0.27	0.21
KS	3.75	1	0.79

*Step 6*: Calculations were made for the pairwise comparison matrix and normalised matrix of criteria with respect to the goal. The criterion–goal matrix is consistent (CR= 0.05 <0.1). The weights of each of the criteria are presented in Table 5. The normalised values were calculated using Table 4. For example, to find the value of $p_{31}$ in Table 5, we first calculated the summation of the first column in Table 4, $\sum_{j=1}^{n}p_{ij}=(1+3+0.2)=4.2$, and then, we calculated $p_{31}=0.2/4.2=0.05$. The same calculations were repeated for the other values in the table.

*Step 7*: The following calculations represent the inner interdependency of the criteria. Table 6 represents the internal interdependency of the main criteria with respect to CH (Main Criteria-CH). The values above the matrix diagonal in Table 6 represent the average values taken from the respondents; other values were calculated in the same manner as described in step 5 (Table 7, Table 8).

**Table 7:** Internal interdependency (Main Criteria-CO) matrix.

**CO**	CH	KS	Weight
CH	1	0.35	0.26
KS	2.83	1	0.74

**Table 8:** Internal interdependency (Main Criteria-KS) matrix.

**KS**	CH	CO	Weight
CH	1	4.62	0.82
CO	0.22	1	0.18

Table 9, Table 10 show the calculations of the normalised relative impact of decision criteria.

**Table 9:** Relative impact of decision criteria.

	CH	CO	KS
CH	1	0.26	0.82
CO	0.21	1	0.18
KS	0.79	0.74	1

**Table 10:** Normalised relative impact of decision criteria.

	CH	CO	KS
CH	0.5	0.13	0.41
CO	0.105	0.5	0.09
KS	0.395	0.37	0.5

Next, the following calculations present the weights of the main solution criteria depending on their inner-interdependencies.

$$w_{\mathrm{criteria}}=\left[\begin{array}{c} \mathrm{CH}\\ \mathrm{CO}\\ \mathrm{KS} \end{array}\right]=\left[\begin{array}{c} 0.5\\ 0.105\\ 0.395 \end{array}\begin{array}{c} 0.13\\ 0.5\\ 0.37 \end{array}\begin{array}{c} 0.41\\ 0.09\\ 0.5 \end{array}\right]\times \left[\begin{array}{c} 0.28\\ 0.64\\ 0.07 \end{array}\right]=\left[\begin{array}{c} 0.25\\ 0.36\\ 0.38 \end{array}\right]$$

It is clear that the key steps for achieving SC resilience are the most important factors for assessing SC solutions. The second significant factor is the concerns of specialists, followed by SC challenges during the pandemic. The internal interdependencies of the main criteria change the weights from 0.28, 0.64, and 0.07 to 0.25, 0.36, and 0.38. Therefore, the changes caused by the inner interdependencies in ranks are clearly observed.

*Step 8*: Calculations were made for the comparison matrix for the local weights of sub-criteria in relation to their main criteria (Table 11, Table 12, Table 13).

**Table 11:** Comparison matrix and local weights for CH.

**CH**	DV	SD	PI	GM	PS	Weight
DV	1	1	1	5	7	0.28
SD	1	1	3	7	5	0.36
PI	1	0.33	1	6	7	0.24
GM	0.2	0.14	0.17	1	4	0.07
PS	0.14	0.2	0.14	0.25	1	0.04
$\lambda_{\max}=5.24$; CI = 0.1; CR = 0.096

**Table 12:** Comparison matrix and local weights for CO.

**CO**	S	C	Q	CC	T	S	CF	RM	O	CS	Weights
S	1	0.67	2	0.5	0.67	0.5	1	0.33	2	0.2	0.06
C	1.5	1	1	2	0.67	1	2	1	2	1	0.1
Q	0.5	1	1	1	0.33	0.5	1	0.33	1	0.5	0.06
CC	2	0.5	1	1	0.2	0.67	0.5	0.2	1	2	0.07
T	1.5	1.5	3	5	1	1	4	0.2	3	1	0.13
ST	2	1	2	1.5	1	1	5	0.17	2	1	0.1
CF	1	0.5	1	2	0.25	0.2	1	0.2	1	0.2	0.05
RM	2.99	1	2.99	5	5	6	5	1	5	3	0.26
O	0.5	0.5	1	1	0.33	0.5	1	0.2	1	0.2	0.04
CS	5	1	2	0.5	1	1	5	0.33	5	1	0.13
$\lambda_{\max}=11.2$; CI = 0.13; CR = 0.089

**Table 13:** Comparison matrix and local weights for KS.

**KS**	AP	CB	SN	DC	RI	Weights
AP	1	2	5	6	7	0.44
CB	0.5	1	4	5	7	0.31
SN	0.2	0.25	1	0.2	1	0.06
DC	0.17	0.2	5	1	3	0.14
RI	0.14	0.14	1	0.33	1	0.05
$\lambda_{\max}=5.43$; CI = 0.1; CR = 0.096						

*Step 9*: The global weight of each sub-criterion was calculated by multiplying the local weight (step 8) by the inner interdependence weight of the criterion (step 7) (Table 14).

**Table 14:** Sub criteria global weights.

Criteria	*w*_*local*_	Sub criteria	*w*_*inner*_	*w*_*global*_
CH	0.25	DV	0.28	0.07
		SD	0.36	0.09
		PI	0.24	0.06
		GM	0.07	0.02
		PS	0.04	0.01
CO	0.36	S	0.06	0.02
		C	0.1	0.04
		Q	0.06	0.02
		CC	0.07	0.03
		T	0.13	0.05
		ST	0.1	0.04
		CF	0.05	0.02
		RM	0.26	0.09
		O	0.04	0.01
		CS	0.13	0.05
KS	0.38	AP	0.44	0.17
		CB	0.31	0.12
		SN	0.06	0.02
		DC	0.14	0.05
		RI	0.05	0.02

*Step 10*: Each expert compared the five alternatives with respect to each sub-criteria and then accumulated the evaluation matrices to obtain the final matrix (Table 15).

**Table 15:**
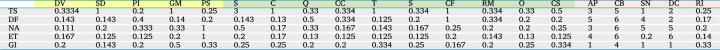
Evaluation matrix.

Then, the normalisation process was performed and the normalised evaluation matrix was computed (equation (9)). The results are shown in Table 16.

**Table 16:**
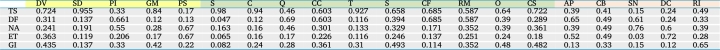
Normalised evaluation matrix.

We then constructed the weighted matrix using the criteria weights from the ANP method and multiplied it by the normalised evaluation matrix using equation (10), as shown in Table 17.

**Table 17:**
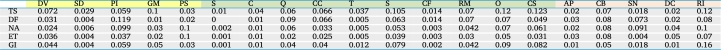
Weighted evaluated matrix.

*Step 11*: We calculated the ideal solution ($v_{i}^{+}$ and $v_{i}^{-}$) using equations (11) and (12). Then, we calculated the Euclidean distance between $S_{i}^{+}$ (positive solution) and $S_{i}^{-}$ (the negative alternative) using equations (15) and (16). After that, the closeness coefficient ($P_{i}$) was computed using equation (17). The results are shown in Table 18.

**Table 18:** Measures of closeness coefficients.

	Si+	Si-	Pi	Rank
**TS**	0.137	0.2	0.593	1
**DF**	0.191	0.151	0.442	3
**NA**	0.161	0.157	0.494	2
**ET**	0.245	0.098	0.286	5
**GI**	0.173	0.134	0.436	4

**Figure 8:**
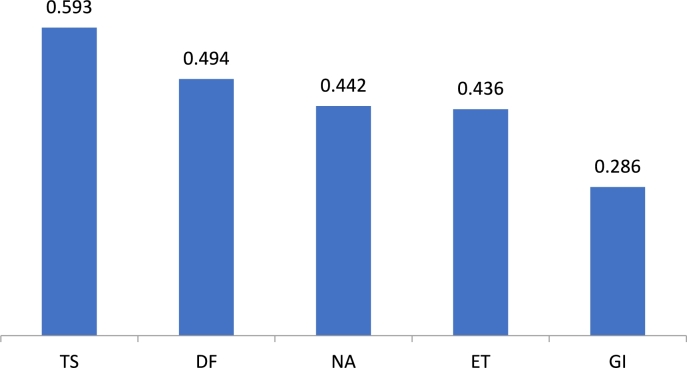
Ranking SC solutions.

*Step 12*: Next, we sorted the final ranks of the solutions based on $P_{i}$ value. The results show that the best solution with the highest rank number is the utilisation of a technological solution followed by integration and network agility (NA). Diversifications of supply came in at rank three, whereas government intervention comes in at four. Empowered team had the lowest (fifth) rank. The ranks for the best SC solutions with COVID-19 in the MENA are shown in Fig. 8.

The results of the proposed framework for the first three solutions are the same as the survey results in phase I (Part IV: Figure [4]). Both agree that technological solutions (TS) are the best alternative followed by diversifications of supply (DF). The rank order of the fourth and fifth alternatives are different; in the framework, the fourth and fifth solutions are empowered team (ET) and government intervention (GI), whereas in phase I they are GI and ET. Thus, the framework is considered valid in selecting the best SC-solutions in the context of the COVID-19 pandemic.

The results obtained are partially expected for such solution rankings as the importance of technological solutions, but surprisingly, it seems that government intervention is less important than the other four solutions. This is because companies consider the negative impact and problems associated with GI as governments suspend work and force companies to pay for employees. Such decisions are characterised by nervousness owing to the unexpected changes that occurring during a pandemic. Small businesses suffer the most from variations in government interventions.

Most companies' SCs in the region were affected by COVID-19 and the related safety measures, including social isolation and distancing, and transportation restrictions. Similarly, most companies lack the contingency plans to manage SC disruptions, and they have experienced sudden, unpredicted delays in receiving goods because of information blackout by SC partners, particularly from sources and suppliers. Many MENA organisations believe that things will return to normal, but lessons learned from this pandemic and previous crises indicate the opposite, mandating immediate action.

ANP can address the interrelationships between decision levels and criteria by obtaining the weights via the supermatrix. TOPSIS is used to rank the solutions to escape additional pairwise comparisons and calculations. TOPSIS methods eliminate several steps performed in ANP and allow the system to reach the outcomes in a shorter time. TOPSIS and ANP are integrated to help organisations to effectively select the best solution and rank order the solutions based on complex interdependencies. The key difficulty is how to build model flexible enough to evaluate all available solutions with respect to the many complex criteria required to make the proposed model a good solution. Furthermore, the model can help managers and executives perform similar multiple-criteria analyses for other SCs and services.

These results of this study answer the research questions and extend the analysis of previous studies to create a fuller picture of the challenges, concerns, and worries generated by COVID-19. It also outlines the key steps for SC sustainability and offers continuing improvements for combatting the effects of COVID-19 and similar future crises.

Previous studies have focused on the impact and challenges of a pandemic or provided a solution for a specific, local, or limited part of the SC. This study, however, identifies appropriate solutions for and the implications of the SC's future stability and performance. Because enterprises cannot adopt every measure at once, these solutions are prioritised based on their significance for and efficacy at limiting the pandemic's impact on the SC, ensuring the flow of commodities, and maintaining future competitiveness. The outcomes can help improve the efficiency of the decision-making process by utilising a framework that combines the ANP and TOPSIS techniques to rank alternative solutions, allowing companies to prioritise the most important SC solutions based on their impact. This real-world case study of the MENA tested the suggested method's logic and applicability. The results indicate that the framework is appropriate for selecting optimal SC solutions in the context of the COVID-19 pandemic.

SCs face many challenges during a pandemic, including availability of liquidity, global disruptions, increased barriers to trade due to government protection measures, personal safety concerns, spikes in demand, and demand changes resulting from shifts in consumers' attitudes. Several steps and factors are concerned with SC sustainability, the new normal, and resilience. Organisations should employ several tools to achieve SC targets, such as by complying with global SC strategy; utilising SC experts to find innovative solutions; assessing, monitoring, and adjust to disruptions; communicating timely with suppliers, partners, and customers; and controlling risk. Whereas most organisations have been negatively impacted, others have found opportunities in the pandemic. The pandemic created many opportunities for organisations to increase the implementation of digital technologies for improving SC resilient and to apply intelligent tools to increase business outcomes.

## 7 Conclusion

The COVID-19 pandemic has had a global impact on all aspects of life. The effect has varied by country, region and product, in addition to the number of infected, government measures, and safety requirements. However, in general, the economy and business have been affected in most countries of the world and in almost all sectors. The most affected sectors have been SCs, especially the GSC, which are the lifelines of the people. The SC has been impacted in an unprecedented way, with disruptions in terms of supply, distribution, and process. Disruptions greatly affect SC operations, and thus the availability of goods to consumers in appropriate quantities, quality, prices, and times.

The organisations realised that there is an urgent need for solutions, whether short-term (like diversification of resources to meet requirements), medium-term (to control prices, quality, and consumer satisfaction), or long-term to ensure competitiveness and stability of the SC to combat future crises. Since organisations cannot implement all solutions at the same time, the need to arrange solutions has emerged based on their relative importance and effectiveness.

To analyse the relationship between the SC and the pandemic, this study was implemented in two phases using real data from relevant organisations and companies as well as the opinions of a group of specialists. The first phase included designing and distributing a survey for a number of organisations in the MENA to explore the connection between COVID-19 and the SC with regard to challenges facing the SC, the interests and sources of concern and worries that decision-makers see as influencing the SC business during the pandemic, the appropriate and necessary steps toward the stability of the SC, and the solutions that can contribute to achieving the objectives of the SC, such as future resilience and competition.

The process of identifying, analysing, and selecting the appropriate solutions and arranging them according to their priorities is a challenge for decision makers in SCs because there are major differences in the size, nature of work, goals, and types of products of different companies as well as the differences in opinions and experiences of decision-makers and executives in these organisations. In addition, the current problem of selecting the best SC solution is complicated because of the non-traditional criteria. In addition, companies need to prioritise solutions due to interdependency of influence factors, large size and complexity of the problem, and the difficulty of implementing them at the same time. Furthermore, solutions depend on the capabilities of companies in terms of cost, time required, and availability of resources and expertise necessary for implementation.

A combined MCDM approach utilising ANP and TOPSIS methods is used to order the alternative solutions necessary to override the pandemic interruptions and improve SC stability and future resilience. The proposed framework improves decision-making efficiency and assists decision-makers in efficiently selecting solutions based on their importance and influence on company. The suggested framework merges ANP-TOPSIS techniques to rank alternative solutions and allow organisations the ability to prioritise their solutions based on their relative importance. ANP considers the interrelationships between the decision levels and criteria by realising the weights via the super matrix. TOPSIS is used to rank the solutions to avoid additional pairwise comparisons and calculations.

The results from phase I and phase II show that the framework is considered suitable for selecting the best SC-solutions when considering the effect of the COVID-19 pandemic. The results indicate that SCs should continuously employ technologies to survive future competition and crises. It is obvious that technology is a common factor that contributes to all solutions. Diversification of supply is an important factor, and SCs are likely to enter a new age of localisation/regionalisation as a result of the pandemic and government intervention. Network agility and integration with partners is vital to increasing SC visibility and automation. The ET and the participation of the work team in developing solutions and decision-making are important factors in improving the SCs and maintaining competitiveness. The government's intervention is necessary and is required in terms of investing and regulating key SC activities.

This study is important for managers, decision makers, and SC practitioners to facilitate and accelerate decision-making processes in light of the pandemic and future crises. In this study, ten companies responded to a questionnaire; but it would be better and preferable to investigate a larger number of companies with different and disparate businesses so that the results could be generalised to the largest possible segment of SC organisations.

The study has several limitations. Because the primary sources of information come from a wide range of industries and businesses, the research was based on the personal opinions and evaluations of respondents and experts; thus, extended validation and evaluation are possible study topics. In order to mitigate disruptions, this study identified five solutions for the SC; other solutions have yet to be identified. Although the case is representative, gathering more responses from other SC enterprises in different places can help to expand the generalisability of the conclusions and overcome the study's limitations. In future research, it is advised that some statistical, simulation, and modelling approaches be combined with the current study. Additional investigations may use other MCDM analysis techniques such as fuzzy-ANP, fuzzy-TOPSIS, ELECTRE, AHP, and PROMETHEE, as well as any combination of these methods.

Future studies should focus on new technological systems. Subsequent research may arrange technological alternatives according to their importance, find a specific mechanism to follow up technological development, and explore the ways to utilise technological solutions in the SCs to ensure steadiness and flexibility against upcoming crises.

## Declarations

### Author contribution statement

Ghazi Mustafa Magableh: Conceived and designed the experiments; Performed the experiments; Analyzed and interpreted the data; Contributed reagents, materials, analysis tools or data; Wrote the paper.

Mahmoud Mistarihi: Performed the experiments; Analyzed and interpreted the data; Wrote the paper.

### Funding statement

This research did not receive any specific grant from funding agencies in the public, commercial, or not-for-profit sectors.

### Data availability statement

Data included in article/supplementary material/referenced in article.

### Declaration of interests statement

The authors declare no conflict of interest.

### Additional information

No additional information is available for this paper.
